# Supplementary material for: High-Glass-Transition Polyesters Produced with Phthalic Anhydride and Epoxides by Ring-Opening Copolymerization (ROCOP)
Source: Polymers (Basel). 2023 Jun 24;15(13):2801. doi: 10.3390/polym15132801 (PMC10347009; doi:10.3390/polym15132801)
Supplement: Supplementary file 1 [file polymers-15-02801-s001.zip › polymers-2418096-supplementary.pdf]

Supplementary Materials

High-Glass-Transition Polyesters Produced with Phthalic Anhydride and Epoxides by Ring-Opening Copolymerization (ROCOP)

Selena Silvano [1,2], Matteo Proverbio [1], Adriano Vignali [1], Fabio Bertini [1,*] and Laura Boggioni [1,*]

[1] Institute of Chemical Science and Technologies—"G. Natta", National Research Council, via A. Corti 12, 20133 Milan, Italy; selena.silvano@scitec.cnr.it (S.S.); matteo.proverbio@outlook.it (M.P.); adriano.vignali@scitec.cnr.it (A.V.)

[2] National Interuniversity Consortium of Materials Science and Technology, INSTM, Via Giuseppe Giusti 9, 50121 Firenze, Italy

* Correspondence: laura.boggioni@cnr.it (L.B.); fabio.bertini@scitec.cnr.it (F.B.)

Figure S1. SEC elution traces of typical A) poly(LO-*alt*-PA) and B) of poly(VCHO-*alt*-PA).

A)

SystemDescription W600+W717+DRI410;THF Stab; 0.6;35°C; Deg

ColumnDescription 3PL (Poli, Oligo, 50A)

SampleDescription Poliestere Boggioni Camp LOPA 77

Vial 8 | Injection 1

Injection Volume 150.00 ul | Run Time 60.00 Minutes

Channel Description 410+32 | Channel 410

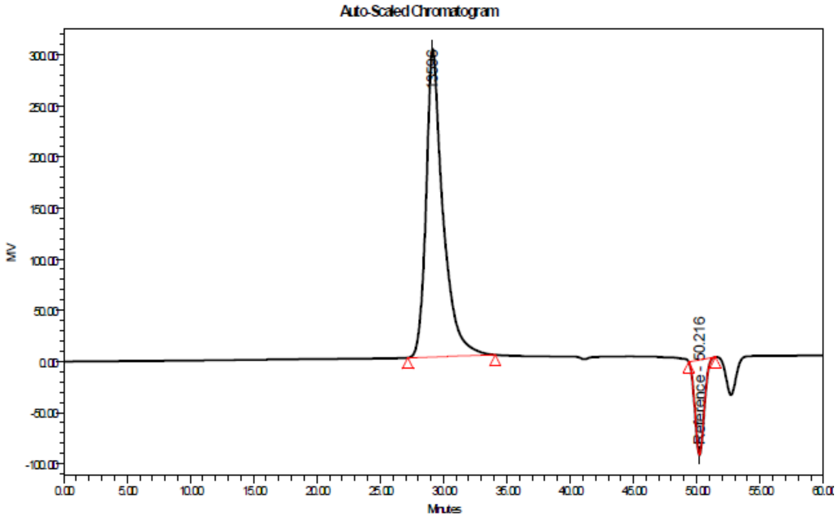

Processed Channel: 410+32

	RT (min)	Area	% Area	Height	% Height	Int Type	SignalNoise
1	29.144	27947146	86.00	301136	76.39	bb	57.160
2	50.216	4550510	14.00	-93047	23.61	bb	17.662

B)

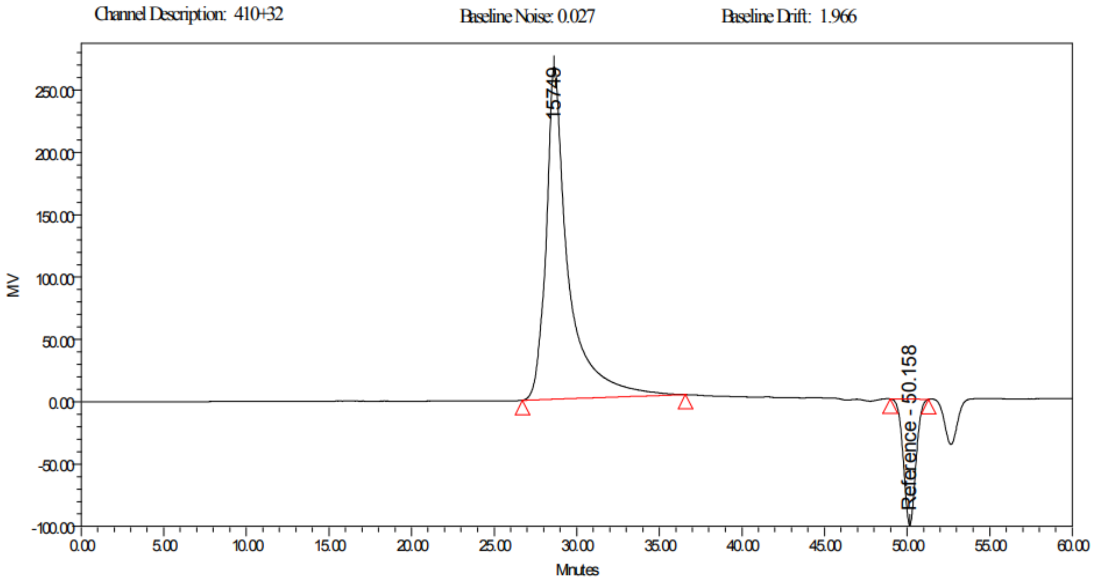

Peak Results

	RT	Area	%Area	Height	%Height	Int Type	SignalNoise
1	28.641	24998694	83.57	267062	72.53	bb	9780.720
2	50.158	4915146	16.43	-101139	27.47	bb	3704.070

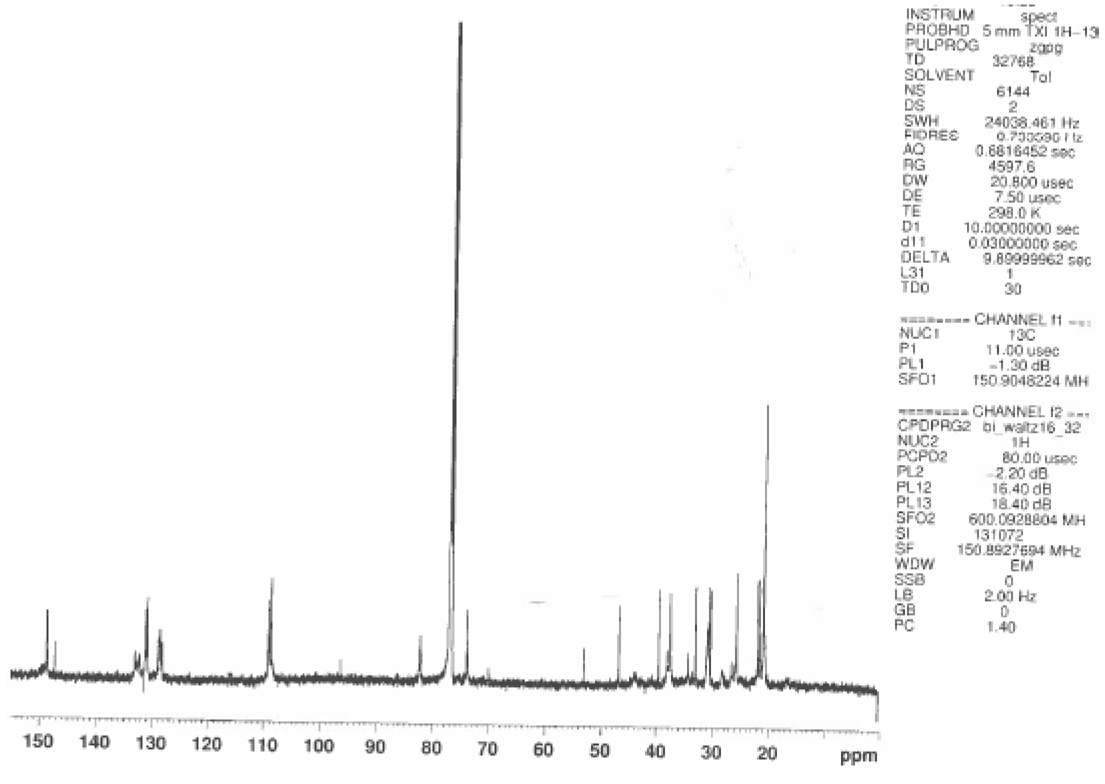

Figure S2. Typical ^{13}C NMR spectrum of a poly(LO-*alt*-PA)

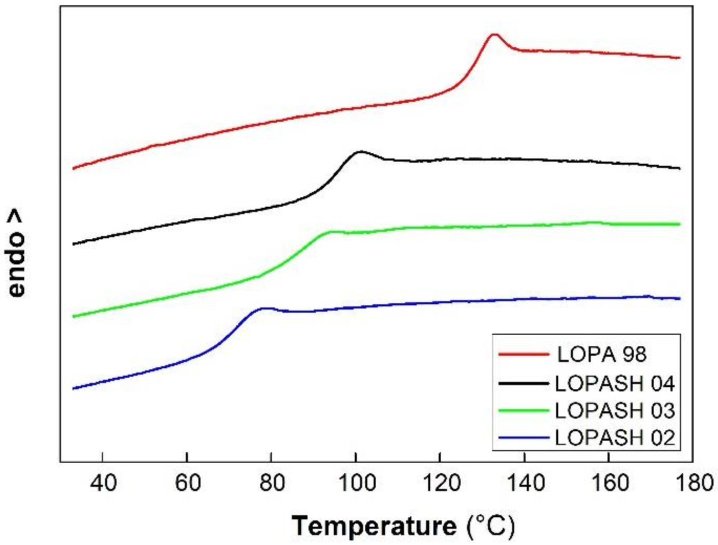

Figure S3. DSC heating scans of LOPA 98, LOPASH 04, LOPASH 03 and LOPASH 02.

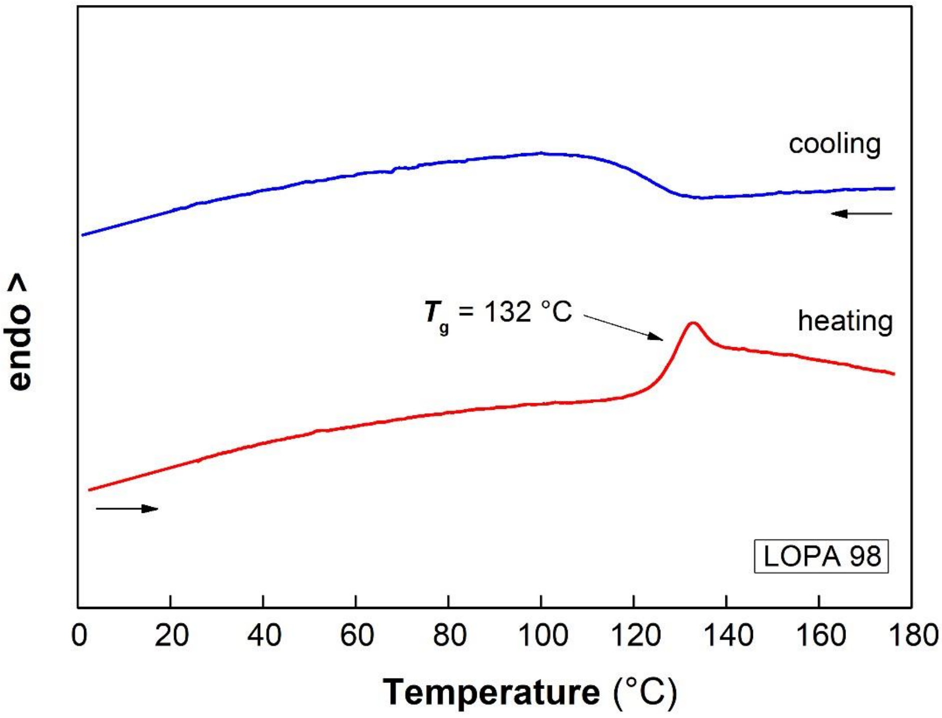

Figure S4. DSC thermal cycle of LOPA 98.

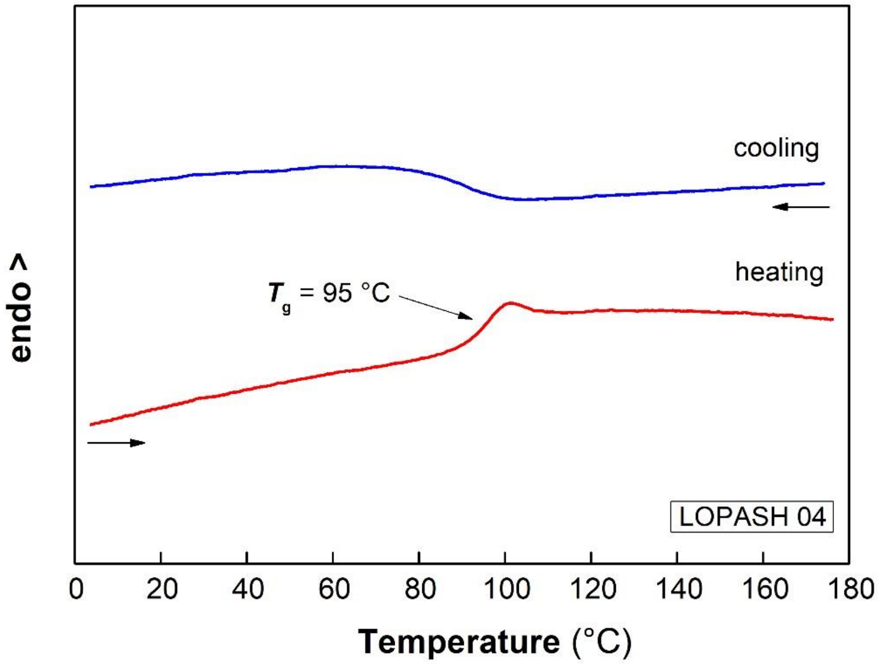

Figure S5. DSC thermal cycle of LOPASH 04.

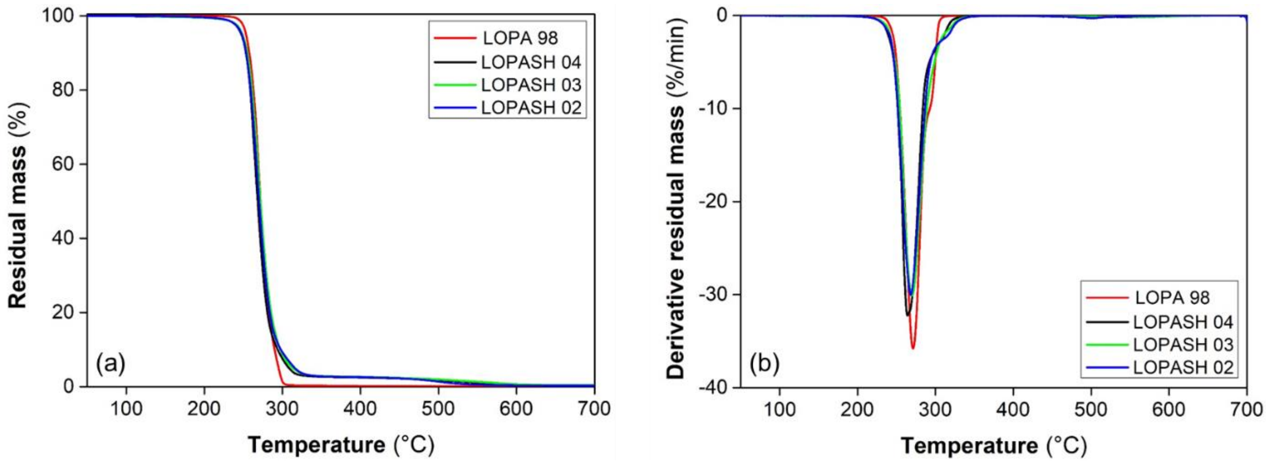

Figure S6. TGA (thermogravimetric analysis) (**a**) and DTG (derivate thermogravimetry) (**b**) curves under nitrogen flow of LOPA 98, LOPASH 04, LOPASH 03 and LOPASH 02.

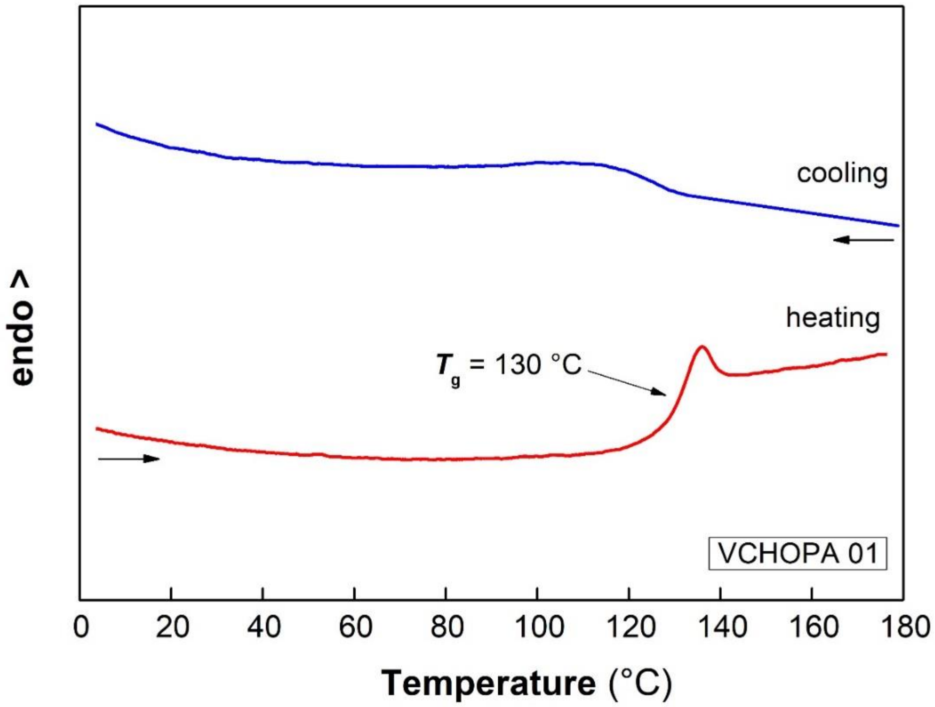

Figure S7. DSC thermal cycle of VCHOPA01.

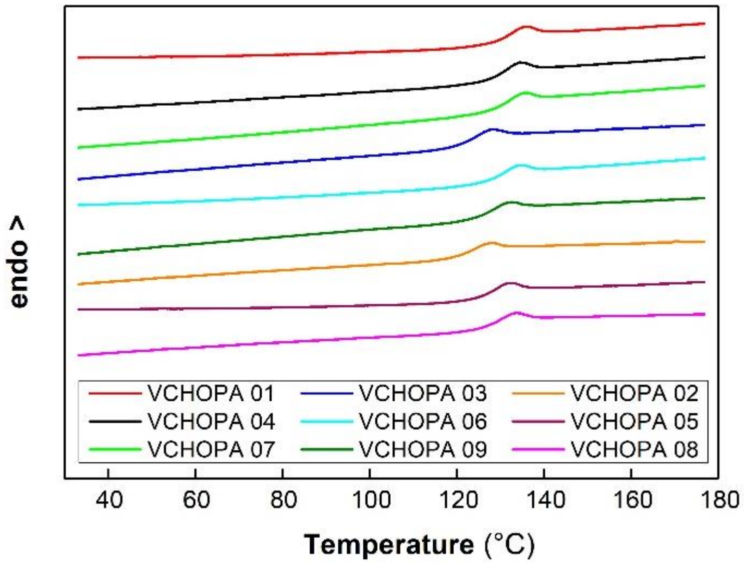

Figure S8. DSC heating scans of poly(VCHO-*alt*-PA) polyesters.
